# Here we go again: More diseases dubiously attributed to pegivirus infection

**DOI:** 10.1371/journal.ppat.1013641

**Published:** 2025-10-28

**Authors:** Adam L. Bailey

**Affiliations:** Department of Pathology & Laboratory Medicine, School of Medicine & Public Health, University of Wisconsin–Madison, Madison, Wisconsin, United States of America; University of North Carolina at Chapel Hill, UNITED STATES OF AMERICA

Pegiviruses (PgVs) are a genus of +ssRNA viruses currently classified within the *Flaviviridae* family [1,2]. Pegivirus infections are widespread in mammals, and two divergent PgVs have been discovered in humans: HPgV-1 (aka. *Pegivirus hominis*), which infects ~15% of the global human population; and the much rarer HPgV-2 (aka. *Pegivirus columbiaense*) [1,2]. These viruses cause high-titer viremia that can persist for years to decades, with the phenomenon of viral clearance being well-documented but poorly understood. Due to the high prevalence of HPgV infection in humans and the abundance of viral RNA in infected individuals, metagenomic studies on human samples routinely detect HPgV infections. Iatrogenic HPgV infections, via procedures like blood transfusion, are presumed to be common but HPgV diagnostics are not used clinically.

PgVs are phylogenetically related to several important human pathogens with hepatitis C virus (HCV) being the closest medically relevant virus, though these viruses share only ~50% nucleotide and ~30% amino acid sequence identity. However, unlike HCV, HPgV has never been shown to play a causal role in any disease. This is not for lack of effort: since the discovery of HPgV, the HPgV field has lurched from one disease association to the next. When HPgV was first identified in 1995 in patients with hepatitis, two different research groups gave it the names “Hepatitis G virus (HGV)” and “GB virus C (GBV-C)” with the assumption that this virus was hepatotropic and responsible for chronic hepatitis [3,4]. Indeed, due to its high prevalence in the human population and its shared modes of transmission with other hepatitis viruses, early studies consistently identified HGV in cohorts of patients with unexplained hepatitis. It took years of careful epidemiological studies comparing healthy and diseased populations to definitively rule out HGV/GBV-C as a cause of hepatitis (summarized in [1,5]), which was further confirmed by prospective longitudinal studies in chimpanzees [6]. Nevertheless, this misconception persists to this day [7]. In the 2000s, several large clinical studies associated HPgV (officially renamed from GBV-C/HGV in 2013) with *improved* outcomes in HIV+ individuals [8]. Since this time, the majority of research on HPgV has focused on understanding the interactions between HPgV and HIV/AIDS disease progression (summarized in [9]). Although some evidence suggests that HPgV may directly inhibit HIV replication (e.g., by reducing HIV receptor/co-receptor expression in CD4 T cells), the mechanism(s) underlying the observed clinical associations remain far from clear. Later, in the 2010s, several studies identified a weak association between HPgV infection and the development of various lymphoid malignancies (see [10] for a meta-analysis), though again, the mechanistic underpinnings of this association remain unclear.

Recently, several case reports and small case-control studies (including one published in the New England Journal of Medicine) have attempted to establish a link between HPgV infection and a spectrum of neurological disorders including encephalitis [11–16], osloclonus-myoclonus [17], optic neuritis and myelitis [18,19], and Parkinson’s disease [20]. Given the prevalence of HPgV, its identification in any population—including patients with neurological diseases—is unsurprising [21]. Nevertheless, a link has been pursued. Imaging and histologic analyses in these studies have revealed nonspecific abnormalities that could be due to any number of nonHPgV-related processes [11,13,15,18,20]. Detection of HPgV replication intermediates or proteins within neural tissues have relied upon techniques with a high potential for generating false-positive results (i.e., strand-specific PCR and/or immunohistochemistry), the results of which have been published without sufficient data on assay optimization, critical controls, quantification, or consideration for typical patterns of viral staining (e.g., cytoplasmic localization of viral RNA) [11,13,20]. Unconvincing arguments have also been built upon phylogenetic analyses that purport “compartmentalization” of HPgV replication within the central nervous system [11–13]. Small sample sizes and a lack of HPgV+ control cases without disease also limit the power of these studies. Altogether, the data show nothing more than what would be expected from a coincidental HPgV infection [11,12].

It remains possible that HPgV infection is associated with a range of diseases, and/or that HPgV may even play a causal role in the development of some diseases. However, the overwhelming evidence collected to date suggests that HPgV is nonpathogenic; thus, changing this consensus requires substantial evidence to the contrary—evidence that, in this author’s opinion, is currently lacking. While some of the recent articles are appropriately reserved when interpreting their findings [14,21,22], notable exceptions include causality-implying statements such as “neurological involvement due to Pegivirus” [19]; the suggestion that HPgV has “neurotropism and a causative role in pegivirus-associated encephalomyelitis” [11]; or the implication that HPgV plays an active role in “alter[ing] brain and and blood immune and transcriptomic profiles” [20]. The creation of terms like “pegivirus-associated encephalomyelitis” [11] now enshrining this tenuous connection via a self-perpetuating snowball effect that is all too common in medical literature.

As physicians and scientists, we must temper our urge to attribute infections to diseases without definitive evidence. This urge is particularly strong for viruses which, by definition, must infect the cells of its host to survive. Premature attribution of disease can have long-lasting harmful effects on a field. Ironically, the HPgV field is already a prime example of this, with rafts of speculative low-quality studies littering the HPgV literature over the past several decades. Ultimately, this has created an “unserious” reputation that discourages innovation and funding. The current disease-oriented culture of scientific funding is partially to blame, which has forced HPgV research to careen from one disease association to the next. However, if we are to move beyond association into the realm of causation, a concerted effort must be made to first understand the basic biology of PgVs (e.g., receptors, susceptible cell types, mechanisms of immune evasion, see **Box 1**). Such information could transform our understanding of how HPgV interacts with the human body. This “first-principles” approach would lay a foundation upon which connections to human diseases could be rationally investigated; however this approach would initially require a significant investment in basic PgV virology. This starts with the painstaking development of new tools for studying the highly novel biology of PgVs including robust cell culture systems, molecular clones, animal models, immunology and protein biochemistry tools, organoids, etc. Although daunting, the success of the HCV) research field—which initially faced many of the same challenges—has proven that this approach can work [23]. A solid foundation of basic PgV biology will ultimately spawn the development of better tools for clinical investigation of HPgV infections. Unfortunately, without such investments, the impact of HPgV infection on human health will likely remain an enigma.

**Box 1. Outstanding questions in pegivirus basic biology**

- What are the cellular receptors(s) utilized by PgVs in general, and HPgV specifically?
- What are the cell types that support PgV replication?
- How does receptor expression influence cellular and tissue tropism?
- How does PgV infection alter host cell viability and function?
- How do PgVs avoid/evade innate and adaptive immune responses?
- In the subset of individuals who clear PgV infection, what are the immunological mechanisms of PgV clearance?

## References

[1] Stapleton JT, Foung S, Muerhoff AS, Bukh J, Simmonds P. The GB viruses: a review and proposed classification of GBV-A, GBV-C (HGV), and GBV-D in genus Pegivirus within the family Flaviviridae. J Gen Virol. 2011;92(Pt 2):233–46. doi: 10.1099/vir.0.027490-0 21084497 PMC3081076

[2] Yu Y, Wan Z, Wang J-H, Yang X, Zhang C. Review of human pegivirus: prevalence, transmission, pathogenesis, and clinical implication. Virulence. 2022;13(1):324–41. doi: 10.1080/21505594.2022.2029328 35132924 PMC8837232

[3] Simons JN, Leary TP, Dawson GJ, Pilot-Matias TJ, Muerhoff AS, Schlauder GG, et al. Isolation of novel virus-like sequences associated with human hepatitis. Nat Med. 1995;1(6):564–9. doi: 10.1038/nm0695-564 7585124

[4] Linnen J, Wages J Jr, Zhang-Keck ZY, Fry KE, Krawczynski KZ, Alter H, et al. Molecular cloning and disease association of hepatitis G virus: a transfusion-transmissible agent. Science. 1996;271(5248):505–8. doi: 10.1126/science.271.5248.505 8560265

[5] Alter HJ. G-pers creepers, where’d you get those papers? A reassessment of the literature on the hepatitis G virus. Transfusion. 1997;37(6):569–72. doi: 10.1046/j.1537-2995.1997.37697335149.x 9191815

[6] Bukh J, Kim JP, Govindarajan S, Apgar CL, Foung SK, Wages J Jr, et al. Experimental infection of chimpanzees with hepatitis G virus and genetic analysis of the virus. J Infect Dis. 1998;177(4):855–62. doi: 10.1086/515255 9534956

[7] Vazzana R, Mularoni A, Vaiana C, Cona A, Mulè G, Amato C, et al. Shotgun metagenomics detects the human pegivirus complete genome in a pediatric patient with acute hepatitis of unknown etiology: a case report. Front Genet. 2025;16:1653082. doi: 10.3389/fgene.2025.1653082 41019762 PMC12460081

[8] Xiang J, Wünschmann S, Diekema DJ, Klinzman D, Patrick KD, George SL, et al. Effect of coinfection with GB virus C on survival among patients with HIV infection. N Engl J Med. 2001;345(10):707–14. doi: 10.1056/NEJMoa003364 11547739

[9] Bhattarai N, Stapleton JT. GB virus C: the good boy virus? Trends Microbiol. 2012;20(3):124–30. doi: 10.1016/j.tim.2012.01.004 22325031 PMC3477489

[10] Fama A, Larson MC, Link BK, Habermann TM, Feldman AL, Call TG, et al. Human pegivirus infection and lymphoma risk: a systematic review and meta-analysis. Clin Infect Dis. 2020;71(5):1221–8. doi: 10.1093/cid/ciz940 31671178 PMC7442854

[11] Scheibe F, Melchert J, Radbruch H, Siebert E, Best TD, Kohler S, et al. Pegivirus-associated encephalomyelitis in immunosuppressed patients. N Engl J Med. 2025;392(18):1864–6. doi: 10.1056/NEJMc2501512 40334164

[12] Bukowska-Ośko I, Perlejewski K, Pawełczyk A, Rydzanicz M, Pollak A, Popiel M, et al. Human pegivirus in patients with encephalitis of unclear etiology, Poland. Emerg Infect Dis. 2018;24(10):1785–94. doi: 10.3201/eid2410.180161 30226156 PMC6154136

[13] Balcom EF, Doan MAL, Branton WG, Jovel J, Blevins G, Edguer B, et al. Human pegivirus-1 associated leukoencephalitis: Clinical and molecular features. Ann Neurol. 2018;84(5):781–7. doi: 10.1002/ana.25343 30246885

[14] Tuddenham R, Eden J-S, Gilbey T, Dwyer DE, Jennings Z, Holmes EC, et al. Human pegivirus in brain tissue of a patient with encephalitis. Diagn Microbiol Infect Dis. 2020;96(2):114898. doi: 10.1016/j.diagmicrobio.2019.114898 31753519

[15] He J, Yang L, Niu C. Human pegivirus detected in patient with reversible severe encephalitis and axillary lymphadenopathy: a case report. Diagn Pathol. 2025;20(1):66. doi: 10.1186/s13000-025-01664-9 40426166 PMC12107937

[16] Fridholm H, Østergaard Sørensen L, Rosenstierne MW, Nielsen H, Sellebjerg F, Bengård Andersen Å, et al. Human pegivirus detected in a patient with severe encephalitis using a metagenomic pan-virus array. J Clin Virol. 2016;77:5–8. doi: 10.1016/j.jcv.2016.01.013 26872326 PMC7106502

[17] Taga A, Kolchinski A, Reed C, Probasco JC, Simner PJ, Green KE, et al. Bridging the gap: opsoclonus-myoclonus syndrome: human pegivirus encephalomyelitis diagnosed through metagenomic next-generation sequencing. Neurology. 2025;105(6):e214091. doi: 10.1212/WNL.0000000000214091 40875928 PMC12599036

[18] Valyraki N, Maillart E, Pourcher V, Shor N, Tran S, Boudot de la Motte M, et al. Human pegivirus identified in severe myelitis and optic neuritis in immunocompromised patients: a pathogenic role for a forgotten virus?. Rev Neurol (Paris). 2023;179(4):361–7. doi: 10.1016/j.neurol.2022.06.011 36302709

[19] Pourcher V, Boudot de La Motte M, Touat M, Deschamps R, Dehais C, Houillier C, et al. Lack of efficacy of sofosbuvir in human pegivirus associated neurological disorders. Rev Neurol (Paris). 2025;181(4):363–4. doi: 10.1016/j.neurol.2025.02.002 40021379

[20] Hanson BA, Dang X, Jamshidi P, Steffens A, Copenhaver K, Orban ZS, et al. Human pegivirus alters brain and blood immune and transcriptomic profiles of patients with Parkinson’s disease. JCI Insight. 2025;10(13):e189988. doi: 10.1172/jci.insight.189988 40626361 PMC12244338

[21] Hardie D, Smuts H. Human pegivirus-1 in the CSF of patients with HIV-associated neurocognitive disorder (HAND) may be derived from blood in highly viraemic patients. J Clin Virol. 2017;91:58–61. doi: 10.1016/j.jcv.2017.04.007 28499138

[22] Carmona R de CC, Cilli A, da Costa AC, Reis FC, Leal É, Dos Santos FCP, et al. Pegivirus detection in cerebrospinal fluid from patients with central nervous system infections of unknown etiology in Brazil by viral metagenomics. Microorganisms. 2023;12(1):19. doi: 10.3390/microorganisms12010019 38257846 PMC10818654

[23] Manns MP, Maasoumy B. Breakthroughs in hepatitis C research: from discovery to cure. Nat Rev Gastroenterol Hepatol. 2022;19(8):533–50. doi: 10.1038/s41575-022-00608-8 35595834 PMC9122245

